# Experiments on the Temperature of Bodies after Death

**Published:** 1846-03

**Authors:** Benjamin Hensley


*Experiments on the Temperature of Bodies after Death.* By Benjamin Hensley, Jr., M. D. (Communicated by Professor Dunglison.)

Marietta, Ohio, January 30, 1846.

*My Dear Doctor:*—Before leaving Philadelphia, I wrote an account of my experiments on *post mortem* heat, intending to leave it with a friend to hand to you; as I left the city quite unexpectedly. I notice this morning, for the first time, that this paper is in my trunk, and hasten to make amends by sending you a copy.

I used the thermometer mentioned by Dr. Dowler, of New Orleans, viz., Réaumur's, composed of a large glass cylinder, with the bulb at one extremity, the large cylinder enclosing a smaller one, and a graduated scale. With an atmospheric pressure of 30 inches of the barometer, the boiling and freezing points were found less than half a degree too low each, and I was assured by the maker that it was correct.

I give you a copy, verbatim, of my notes, that you may draw your own conclusions. I will further state that I made these experiments myself, and *know* they are scrupulously correct, so far as my agency is concerned. I cannot, of course, pretend to say they might not have been affected by manipulation and other causes, over which I had no control. In the notes below, the *time* the thermometer is retained in a region is first given, generally fifteen minutes (15′), and following this the number of degrees and minutes in Réaumur's scale.

The first case was an infant 10 months old. Three hours before death.—Temperature of room 19° R. Axilla, fifteen minutes (15′), twenty-three degrees (23°), thirty minutes (30′). Hand, 15′ 24° 30′. Fifty minutes after death.—Temperature of room 18°. Axilla, 15′ 23° 30′. Perineum, 25′ 22° 45′. Axilla, 25′ 23° 15′. Popliteus, 20′ 23°. Bend of arm, 20′ 22° 45′. Axilla, 20′ 22° 30′. Vagina, 15′ 21° 45′. Popliteus, 15′ 21° 45′. Bend of arm, 25′ 22° 30′. Perineum, 15′ 21° 15′. Axilla, 15′ 21° 45′. Vagina, 15′ 21°. Experiments closed.—Temperature of room 17° 30′.

Experiments resumed, 16 hours after death.—Temperature of room 17° 30′. Axilla, 15′ 17° 30′. Vagina, 15′ 16° 45′.

Nineteen hours after death.—Temperature of room 16°. Bend of arm, 15′ 17°. Axilla, 15′ 17°. Vagina, 15′ 16° 30′.

Thirty-six hours after death.—Temperature of room 14°. Axilla, 15′ 14°. Vagina, 15′ 13° 30′. Experiments now closed; signs of decomposition.

---

Case 2.—A man died of old age, (80 years.) 3 hours 40 minutes after death.—Temperature of dead-house 17° R. Axilla, 20′ 25°. Rectum, 15′ 27° 30′. Pharynx, 15′ 24° 15′. Popliteal region, 15′ 19° 30′. Bend of arm, 15′ 23°. Axilla, 15′ 23° 45′. Rectum, 15′ 26° 15′. Pharynx, 15′ 23° 30′. Bend of arm, 15′ 22°. Experiments now closed.—Temperature of room, 17°.

Eleven and a half hours after death.—Temperature of dead-house 13° 30′. Axilla, 15′ 21° 30′. Rectum, 15′ 22°. Bend of arm, 15′ 20°.

Thirty-one hours after death.—Temperature of room 16°. Axilla, 15′ 16°. Rectum, 15′ 16°. Experiments now closed: subject emaciated.

Case 3.—A man aged 40, died of cardiac disease, complicated with general dropsy.—Temperature of room 14°.

Thirteen hours after death.—Brain, 15′ 19° 15′. Axilla, 15′ 22° 30′. Scroto-inguinal region, 15′ 24° 30′. Between malleoli, 15′ 16°. Between left pleura, 15′ 24°. Thigh, (a deep incision,) 15′ 21° 45′. Brain, 15′ 18° 45′. Axilla, 15′ 21° 45′. Between left pleura, 15′ 23°. Scroto-inguinal region, 15′ 23° 15′. Thigh, 15′ 20° 45′. Between malleoli, 15′ 15° 15′. Scroto-inguinal region, 15′ 22° 45′. Experiments now closed.—Temperature of room 16°.

Thirty-six hours after death.—Temperature of dead-house 14°. Brain, 15′ 13° 45′. Between left pleura, 15′ 16° 15′. Scroto-inguinal region, 15′ 16°. Thigh, (old incision,) 15′ 14° 30′. Axilla, 15′ 16°. Between malleoli, 15′ 13° 30′. Experiments now closed.

This man is very much puffed up with water; decomposition has commenced; (fetor quite perceptible.) I notice local difference of temperature in the room; at the feet the thermometer stands at 13° 30′, while, up about the abdomen, it rises to full 14°.

Case 4.—A colored man, aged 50, died of diarrhœa. Temperature of dead-house 15° R.

Two and a half hours after death. Axilla, 15′ 27° 15′. Scroto-inguinal region, 15′ 26° 15′. Axilla, 15′ 27°. Between malleoli, 15′ 21°. Hand, 15′ 19° 15′. Brain, 15′ 24°. Between left pleuræ, 15′ 26°. Axilla, 15′ 25°. Thigh, 15′ 23°. Scroto-inguinal region, 15′ 24°. Temperature of room 15° 30′. Experiments now closed.

Nine hours after death.—Temperature of room 14° 30′. Brain, 15′ 20° 45′. Between left pleuræ, 15′ 25°. (In all cases the incisions were made valvular.) Thigh, (new incision,) 15′ 19° 30′. Between malleoli, 15′ 16° 30′. Scroto-inguinal region, 5′ 20° 45′. Axilla, 5′ 23°. (In my observations of the conduct of the thermometer, which were numerous, it always rose to the maximum in less than five minutes.)

Twenty-seven and a half hours after death.—Temperature of dead-house 14° 30′. Brain, (new opening,) 15′ 16° 30′. Between right pleuræ, (new opening,) 15′ 19° 45′. Scroto-inguinal region, 15′ 16° 45′. Malleolus, 15′ 14° 30′. Thigh, (new incision,) 15′ 15° 15′. Axilla, 15′ 18° 30′. Experiments now closed.

Case 5.—A boy, aged 16. One hour 45 minutes after death.—Temperature of dead-house 14° 30′. Axilla, 15′ 28°. Rectum, 15′ 30°. Popliteus, 15′ 24°. Axilla, 15′ 27° 30′. Bend of arm, 15′ 24°. Rectum, 15′ 29°. Brain, 15′ 24° 15′. Experiments now closed.

Seventeen and a half hours after death.—Temperature of room 14° 30′. Brain, (new opening,) 15′ 16°. Rectum, 15′ 19°. Axilla, 15′ 17° 45′. Taken away by friends.

Case 6.—A woman, aged 35, died of mania-a-potu. Ten minutes after death.—Temperature of room 15° R. Axilla, 15′ 27° 45′. Popliteus, 15′ 25° 45′. Removed to dead-house.

One hour and twenty minutes after death.—Temperature of dead-house 15°. Axilla, 15′ 25° 15′. Vagina, 15′ 29°. Experiments now closed.

Sixteen hours after death.—Temperature of dead-house 14° 30′. Axilla, 15′ 18°. Vagina, 15′ 19° 15′. Removed by friends.

Case 7.—A man, aged 36. Thirty minutes after death—Temperature of ward 18°. (This man died of ascites following prolonged intermittent. He was very much swollen. Indeed, the dropsy was general, as the whole subcutaneous cellular tissue was infiltrated.) The axilla gave in 15′ 27° 30′. Scroto-inguinal region, 15′ 28° 30′, (very much inflamed from dropsical infiltration.) Malleolus, 15′ 23° 15′. Removed to dead-house.

Thirty minutes afterwards.—Temperature of dead-house 15°. Axilla, 15′ 27°. Rectum, 15′ 28°. Thigh, 15′ 27°. Bend of arm, 15′ 24° 30′. Brain, 15′ 24°. Between malleoli, 15′ 20°. Scroto-inguinal region, 15′ 27°.

Seventeen hours fifteen minutes after death.—Temperature of dead-house 14° 30′. Scroto-inguinal region, 20′ 18° 15′. Brain, 15′ 15° 15′. Axilla, 15′ 19°. Rectum, 15′ 20° 30′.

Twenty-six hours after death.—Temperature of dead-house 17° 30′. Axilla, 15′ 17° 45′. Brain, 15′ 14° 30′. Rectum, 15′ 18°. Scroto-inguinal region, 15′ 16° 30′. Experiments closed.

Case 8.—A woman, aged 36, died of chronic dysentery; is emaciated.

Four hours and fifteen minutes before death.—Temperature of room 18° R. Axilla, 15′ 27° 45′. Hand, 15′ 24°. Popliteus, 15′ 27°. One minute post-mortem.—Temperature of ward 20°. Axilla, 15′ 27° 45′. Popliteus, 15′ 26°. Axilla, 15′ 27°.

Two hours and twenty minutes after death.—Temperature of dead-house 19° R. Brain, 15′ 24° 30′. Vagina, 15′ 26° 30′. Between left pleuræ, 15′ 25° 30′. Between diaphragm and liver, 15′ 25° 30′. Axilla, 15′ 23° 45′. Popliteus, 15′ 20° 30′.

Eighteen hours, post-mortem.—Temperature of dead-house 17°. Between right pleuræ, (new opening,) 15′ 17° 45′. Rectum, 15′ 18° 30′. Brain, (new opening,) 15′ 17°. Experiments closed.

Case 9.—A woman died of old age, (85 years;) very much emaciated.

Two hours after death.—Temperature of dead-house 18° R. Axilla, 15′ 28° 45′. Popliteus, 15′ 25° 30′. Axilla, 15′ 28° 15′.

Six hours after death.—Temperature of dead-house 19° R. Brain, 15′ 24°. Vagina, 15′ 28°. Between left pleuræ, 15′ 26°. Between liver and diaphragm, 15′ 26° 15′. Rectum, 15′ 27°. Experiments closed.

P. S.—I refer you particularly, in the above paper, to the difference of temperature in the brain and other regions.

I am, dear Doctor,

Truly yours,

Ben. Hensley, Jun.

Professor Dunglison.

